# Partnering With Caregivers to Optimize Hospital-Based Geriatric Care—The Time Is Now

**DOI:** 10.1093/geroni/igaf122.036

**Published:** 2025-12-31

**Authors:** Beth Fields, Lauren Bangerter

## Abstract

A growing number of family caregivers (relatives, partners, or other support persons) are providing support to older adults during hospital stays. However, many of these caregivers report feeling unrecognized and disengaged in hospital care processes. To address this gap, AARP championed the Caregiver Advise Record and Enable (CARE) Act, now law in 45 states and US territories. This law mandates that hospitals: 1) record the name of a family caregiver in the patient’s healthcare record, 2) inform the family caregiver when the patient is to be discharged, and 3) provide the family caregiver with education on tasks to be performed for the patient. In response to this legislation, this symposium will present innovative approaches to enhance partnership with family caregivers in hospital settings. The information presented aims to bridge the policy and practice gap by offering healthcare clinicians, administrators, and researchers practical examples of when, where, why, and how to involve family caregivers in hospital care. The first presentation will describe the role of caregiver feedback in the adaptation of a caregiver support intervention for hospital care. The second presentation will discuss challenges and strategies for identifying family caregivers in the hospital setting. The third presentation will report findings from implementing a caregiver readiness for hospital discharge assessment. The fourth and final presentation will share a co-designed toolkit for including and supporting family caregivers at the system level.

